# A sellar mass as the initial presentation of pituitary infiltration by diffuse large B-cell lymphoma: a case report

**DOI:** 10.3389/fonc.2026.1686394

**Published:** 2026-04-21

**Authors:** Gengxue Jin, Xiangyu Xu, Huitao Meng, Jing Liu, Hui Chen

**Affiliations:** 1 Department of Endocrinology and Metabolism, Lanzhou University Second Hospital, Lanzhou, Gansu, China; 2 Department of Pediatric Cardiology, Lanzhou University Second Hospital, Lanzhou, Gansu, China; 3 Department of Endocrinology and Metabolism, Lanzhou Honggu District People’s Hospital, Lanzhou, Gansu, China

**Keywords:** adrenal insufficiency, diffuse large B-cell lymphoma, pituitary gland, pituitary infiltration, sellar mass

## Abstract

Systemic lymphoma involving the pituitary region is rare and often presents with nonspecific symptoms, making diagnosis challenging. This work reports a case of diffuse large B-cell lymphoma (DLBCL) involving the pituitary gland that initially appeared as a sellar mass. The clinical presentation, diagnostic workup (including biochemical and imaging findings), and management of this condition were discussed.

## Introduction

Pituitary infiltration by hematologic malignancies is rare (1), and systemic lymphoma is the primary cause, accounting for approximately 0.5% of these cases (2, 3). Diffuse DLBCL, the most common subtype of non-Hodgkin lymphoma (NHL), is characterized by aggressive clinical behavior and remarkable biological heterogeneity. A subset of patients develops refractory or relapsed disease after first-line therapy, resulting in significantly shortened survival (4).

The clinical manifestations of pituitary infiltration from DLBCL exhibit remarkable heterogeneity. The diagnosis and management of these patients present a complex clinical challenge that requires a dedicated multidisciplinary team approach. Therefore, we present a case of pituitary infiltration that initially manifested as a sellar mass to enhance clinicians’ recognition of this condition and facilitate early intervention.

## Case description

**Figure 1:**
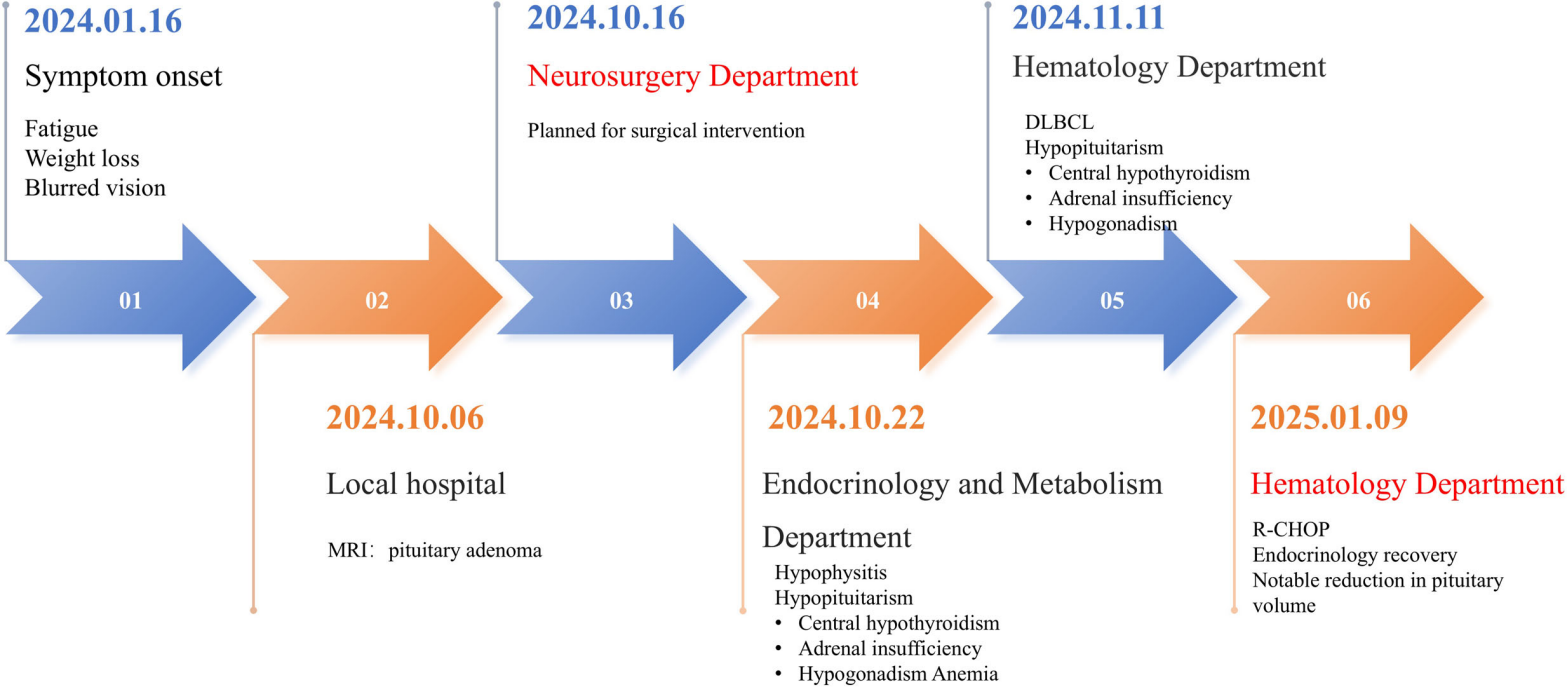
Patient visit experience.

A 54-year-old female presented to the Lanzhou University Second Hospital on October 24, 2024, with a 10-month history of fatigue and progressive weight loss. Ten days prior to admission, a brain magnetic resonance imaging (MRI) at a local hospital for blurred vision revealed findings consistent with a pituitary adenoma. After surgical consultation, the patient was admitted to our neurosurgery department for definitive management. The patient visit experience is presented in **Figure 1**.

**Figure 2:**
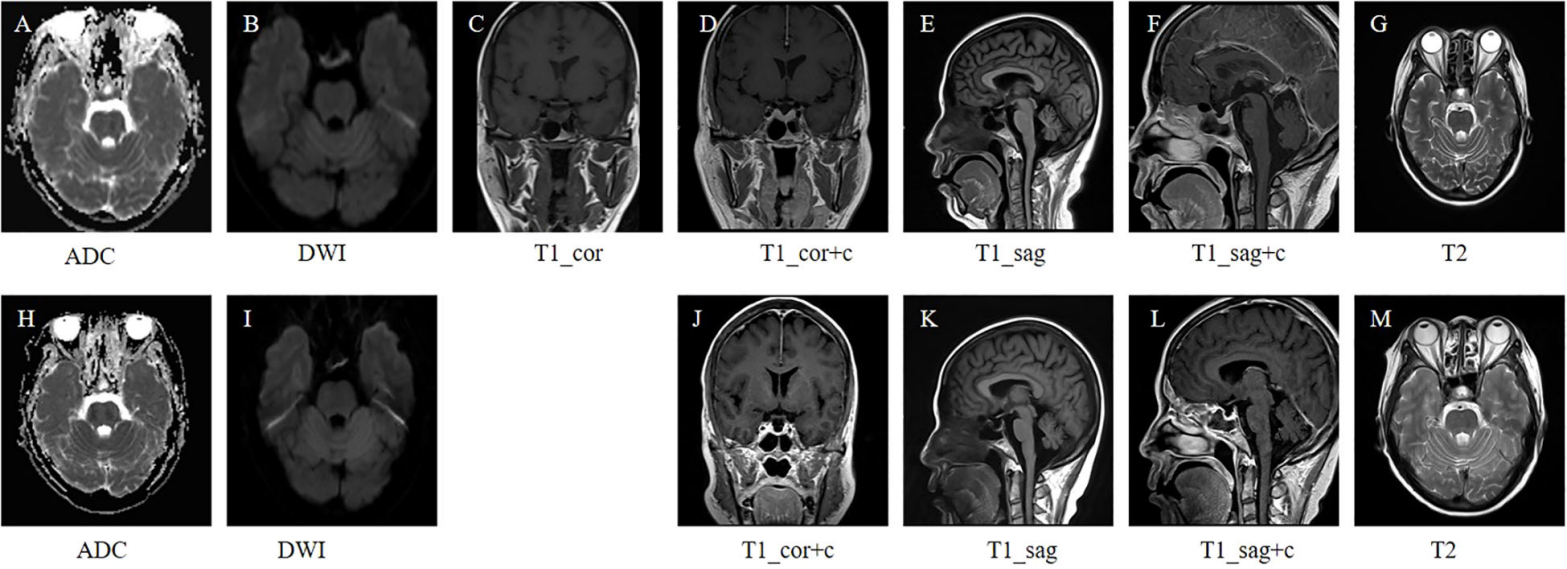
Comparative pituitary MRI scans before and after treatment. **(A-G)** Pre-treatment pituitary MRI revealed glandular enlargement, stalk thickening, and marked enhancement-features most consistent with an inflammatory etiology-with preservation of the neurohypophyseal bright spot (2024.10.28); **(H-M)** Pituitary MRI after two treatment cycles revealed a reduction in pituitary volume and mild thickening of the pituitary stalk, indicating improvement compared to the previous scan (2025.1.13). ADC, Apparent Diffusion Coefficient; DWI, Diffusion-Weighted Imaging: T1_cor, T1-weighted coronal image; T1_sag, T1- weighted sagittal MRI; T1_sag+c, T1-weighted contrast-enhanced sagittal; T2, Transverse relaxation time.

Post-admission contrast-enhanced pituitary MRI showed glandular enlargement, stalk thickening, and marked enhancement, most consistent with an inflammatory etiology, with preservation of the neurohypophyseal bright spot (**Figure 2**). Hormonal evaluation (**Table 1**) revealed anterior hypopituitarism (central hypothyroidism (HT), hypogonadism (HG), growth hormone deficiency (GHD), and adrenal insufficiency (AI)) without hyperprolactinemia (HP) or diabetes insipidus (DI). The patient concurrently presented with pancytopenia, hepatosplenomegaly, and intermittent fever, accompanied by mildly elevated inflammatory markers. Bone marrow biopsy revealed lineage hyperplasia (granulocytic and megakaryocytic) with erythroid predominance, characterized by predominantly polychromatophilic and orthochromatic normoblasts. Physical examination detected left submandibular lymphadenopathy. Ophthalmic examination revealed the following findings: (1) Visual acuity: Uncorrected vision was 0.8 in both eyes (OD and OS). With correction, it improved to 1.0 in both eyes. (2) Intraocular pressure: 11.8 mmHg in the right eye (OD) and 15.6 mmHg in the left eye (OS). (3) Anterior segment: Both corneas were clear. The anterior chambers were of normal depth and clear. The lenses were transparent in both eyes. Fundus examination: The optic disc margins were distinct but pale in both eyes. The foveal reflex was well-defined. A cotton-wool spot was observed in the posterior pole of the right eye. Visual field test: Visual field defects were present in both eyes. Optical coherence tomography of the optic nerve head: The average retinal nerve fiber layer thickness was 114 µm in the OD and 111 µm in the OS. The average cup-to-disc ratios were 0.31 (OD) and 0.24 (OS).

**Table 1:** Clinical and biochemical characteristics of cases reported at diagnosis.

Variable	Baseline (2024-10-16)	3weeks (2024-12-18)	6weeks (2025-1-9)	12weeks (2025-3-26)	Unit	Reference range
FSH	1.38	14.1	-	17.99	mIU/mL	23-116.3
LH	0.1	5.47	-	6.25	mIU/mL	15.9-54
FT4	7.42	-	15.5	14.35	pmol/l	10.44-24.38
TSH	0.097	1.253	0.488	1.391	µU/mL	0.38-4.34
COR	14.85	6.87	-	4.34	µg/dL	5-25
ACTH	1.32	14.9	-	7.49	pg/mL	5-46
PRL	20.02	9.71	-	12.04	ng/mL	2.8-29.2
GH	0.54	0.47		0.18	ng/mL	0-5
IGF-1	38.5	98.8		99.7	ng/mL	31-323
MRI finding	glandular enlargement, stalk thickening, and marked homogeneous enhancement, neurohypophyseal bright spot	-	reduction in pituitary volume and mild thickening of the pituitary stalk, indicating improvement compared to the previous scan	-	-	-
CSF-GLU	4.73	2.8	-	3.33	mmol/l	2.5-4.4
CSF-CL	119.1	122.7		125.5	mmol/l	120-130
PET/CT	hypermetabolic lesions with diffuse metabolism (mediastinal blood pool SUVmax 2.0, liver SUVmax 3.6) on the bilateral parapharyngeal, cervical, and anterior chest subcutaneous lymph nodes (suggestive of lymphomatous involvement), with concomitant focal pituitary hypermetabolism	-	-	a Deauville score of 2	-	-

FSH represents follicle stimulating hormone, LH represents luteinizing hormone, FT4 represents free, TSH represents thyroid stimulating hormone, COR represents cortisol, ACTH represents adrenocorticotropic hormone, PRL represents pituitary prolactin, GH represents growth hormone, IGF-1 represents insulin-like growth factor-1, CSF represents cerebrospinal fluid, PET/CT represents positron emission tomography/computed tomography.

**Figure 3:**
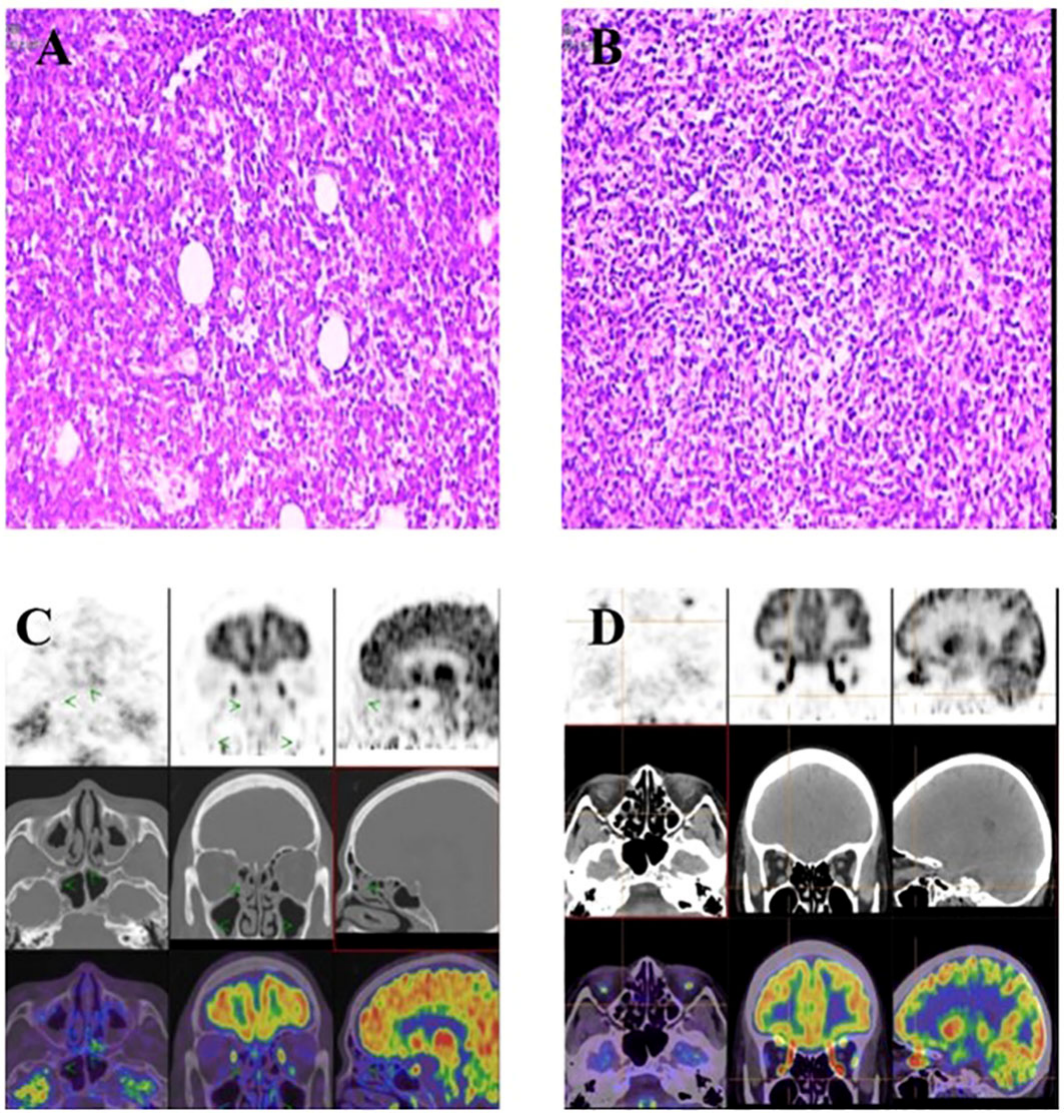
Lymph node case images of the patient and comparative PET/CT scans before and after treatment. **(A, B)** show the pathological results of the cervical lymph node biopsy (2024.11.1); **(C)** shows PET/CT imaging (2025.1.13); **(D)** shows PET/CT imaging after 12weeks treatment (2025.3.27).

Biopsy from left submandibular lymphadenopathy confirmed high-grade B-cell lymphoma, most consistent with non-Hodgkin diffuse DLBCL[CD20+, Pax-5+, CD10 (30%, weak+), MUM-1 (20%, weak+), CD79α+, CD30−, cyclin D1-, CD5 (40%+), BCL-6 (10%, weak+), BCL-2 (50%+), p53 (wild-type expression), CD43-, CKp-, and Ki-67 (90%+)] (**Figure 3**). The patient did not undergo a pituitary gland biopsy.

Positron emission tomography-computed tomography (PET/CT) showed hypermetabolic lesions with diffuse metabolism (mediastinal blood pool SUVmax 2.0, liver SUVmax 3.6) on the bilateral parapharyngeal, cervical, and anterior chest subcutaneous lymph nodes (suggestive of lymphomatous involvement), with concomitant focal pituitary hypermetabolism (**Figure 3**).

The patient was treated with endocrine hormone replacement therapy, including levothyroxine and hydrocortisone. However, the patient voluntarily discontinued the medication one month after discharge without experiencing significant discomfort. Additionally, the patient underwent R-CHOP chemotherapy (rituximab 660 mg, cyclophosphamide 1.3 g on day 1, doxorubicin 80 mg on day 1, vincristine 2 mg on day 1, and prednisone 100 mg on day 1-5) and received five intrathecal injections (methotrexate 12.6 mg, dexamethasone 5 mg, and cytarabine 35 mg). Doses were adjusted based on tolerance, with no significant adverse events.

Following one cycle of chemotherapy, hormone levels normalized. Contrast-enhanced MRI of the pituitary gland after two treatment cycles revealed a reduction in pituitary volume and mild thickening of the pituitary stalk, indicating improvement compared to the previous scan (**Figure 2**). The post-four-cycle PET/CT scan demonstrated a Deauville score of 2, indicating a complete metabolic response following lymphoma treatment (**Figure 3**).

## Discussion

DLBCL, the most prevalent form of non-Hodgkin lymphoma, is a systemic, multifocal, and highly aggressive malignancy with marked heterogeneity (4). It commonly affects both lymph nodes and extranodal organs, though identifying its precise primary site can be challenging. Involvement of the pituitary gland is exceedingly uncommon and is predominantly documented in case reports. Due to the difficulty in determining the origin of the disease, describing pituitary lesions as “infiltration” rather than “metastasis” is generally recommended unless definitive evidence suggests otherwise.

Only 19 cases of pituitary infiltration by DLBCL have been reported to date (**Table 2**) (5–21). The age range of patients with pituitary infiltration by diffuse DLBCL is 39 to 77 years, with no significant gender difference. Our patient, a 54-year-old female, falls within this reported age range, which is consistent with the demographic characteristics of this condition.

**Table 2:** Literature review of previously reported cases (n = 19).

No.	Reference/Y [ref]	Age/Sex	Primary symptoms	Endocrinological features	MRI findings	Follow-up
1	Li/1998 (5)	77/M	Weakness, confusion, polyuria	HT, AI	Pituitary mass	Died 9 wk after diagnosis
2	Mathiasen/2000 (6)	65/M	Low libido, fatigue, weakness, dyspnea	HP, HT, AI, HG	DEPG	Not reported
3	Büchler/2002 (7)	69/F	Weakness, fever, WL, polyuria/polydipsia, anasarca	HP, HT, AI, HG	DEPG	CR after 2nd cycle of CHOP
4	Ogilvie/2005 (8)	59/M	Headache, ptosis, photophobia	HT, AI, HG	Leptomeningeal mass	CR for 18 m Hypopituitarism persists
5	Ogilvie/2005 (8)	53/M	Polyuria, WL, headache, night sweats	HG, DI	ANSPP	Lost to follow-up
6	Kenchaiah and Hyer/2011 (9)	65/F	Lethargy, appetite loss, edema	HP, HT, AI, HG	None PET scan–pituitary	CR, ER
7	Valeros and Khoo/ 2014 (10)	69/M	Dizziness, WL, strabismus, fever, postural hypotension	HT, AI, HG	Hypodensities in pituitary gland	DSS after 2 cycles of CTX
8	Koiso/2014 (11)	78/F	Diplopia, ptosis, back pain, fever	DI	Sellar mass extending to sphenoid and cavernous sinus	CR 4 y after diagnosis
9	Kumabe/2015 (12)	72/F	Anasarca	HT, HG	Swelling of pituitary gland	CR, ER
10	Ravnik/2016 (13)	60/M	Fatigue, WL, night sweats, abdominal pain, nausea/vomiting	HT, AI, HG, DI	Sellar and suprasellar mass and COC	RMRI at 3 y
11	León-Suárez/2016 (14)	64/F	Dyspepsia, nausea/ vomiting thirst, fatigue, WL	HP, HT, AI, HG, DI	Pituitary enhancement, TPS, OC, and HPI	Reduction of TPS, DSS
12	Stegink/2019 (15)	39/M	Massive gastrointestinal bleeding, polyuria	HT, AI, DI	TPS	No response to treatment
13	Jaiswal/2019 (16)	42/F	Amenorrhea, headache, visual field reduction, fatigue, WL, limb pain	PH	Sellar and suprasellar mass. HPI	Died 2 mo after CTX initiation
14	Vega/2021 (17)	69/M	Weakness, hypercalcemia, hypoglycemia	HP, HT, AI	TPS and DEPG	Died 5 d after diagnosis
15	Tovar-Méndez/2022 (18)	60/F	headache, drowsiness, excessive thirst,nausea, and vomiting	DI, HT, AI, HG	Pituitary enhancement, thickened infundibulum with heterogeneous signal	Died of chemotherapy-related side effects a few weeks later.
16	Tovar-Méndez/2022 (18)	44/M	diffuse headaches and generalized tonic– clonic seizures	DI, HT, AI	Heterogeneous anterior pituitary enhancement (no focal lesion); absent posterior pituitary bright signal on T1; intra-axial, supratentorial location	Died after 1 month
17	Alghzawi/2024 (19)	57/F	fatigue, nausea, and anorexia	panhypopituitarism	a partially empty sella with marked thickening and enhancement of the infundibulum with restricted diffusion	Died after 1 month
18	Akkas/2013 (20)	68/M	None	None	Pituitary mass	None
19	Javanbakht/2018 (21)	72/F	None	Visual involvement, HT	None	Died after 36 months

AI, adrenal insufficiency; ANSPP, absence of normal signal from posterior pituitary; CHOP, cyclophosphamide, doxorubicin, vincristine, prednisolone; COC, compression of the optic chiasm; CR, complete response; CT, computed tomography; CTX, cyclophosphamide; DEPG, diffuse enlargement of the pituitary gland; DI, diabetes insipidus; DSS, died of septic shock; ER, endocrinology recovery; HG, hypogonadism; HL, Hodgkin lymphoma; HP, hyperprolactinemia; HPI, hypothalamus infiltration; HT, hypothyroidism; LPL, lymphoplasmacytic lymphoma; MRI, magnetic resonance imaging; NHL, non-Hodgkin lymphoma; NK, natural killer; OC, optic chiasm; PET, positron emission tomography; PH, panhypopituitarism (unspecified pituitary axes); RMRI, reduction on magnetic resonance imaging scan; TPS, thickened pituitary stalk; WL, weight loss.

Approximately 90% of pituitary infiltration by lymphoma cases are attributed to non-Hodgkin lymphoma. Among these, diffuse large B-cell lymphoma (DLBCL) is the most common subtype, representing approximately 50% of cases, followed by Burkitt lymphoma at around 10% (19). In the present case, the aetiology of pituitary infiltration was DLBCL.

**Figure 4:**
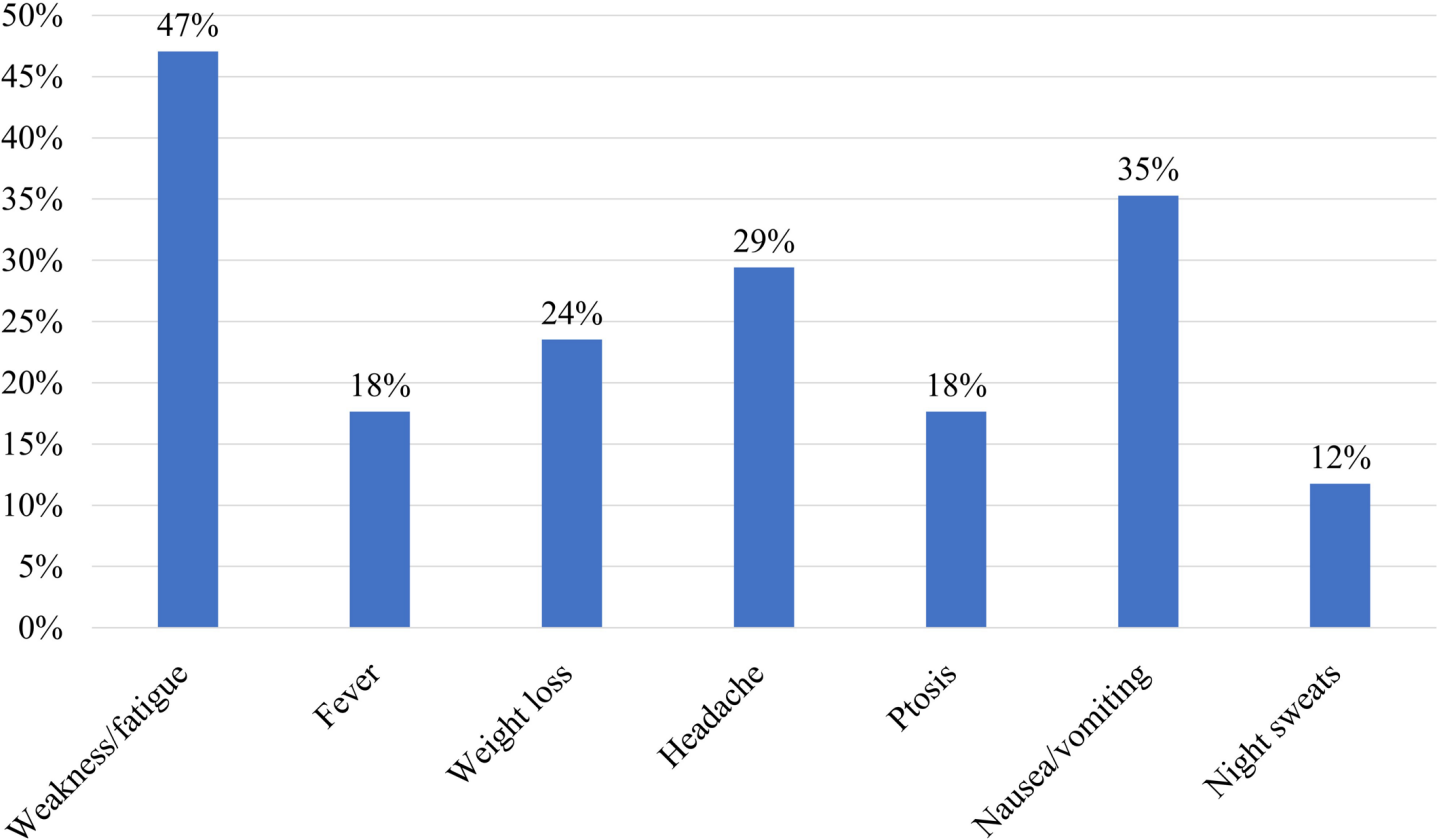
Symptom frequency in patients with pituitary infiltration by DLBCL.

**Figure 5:**
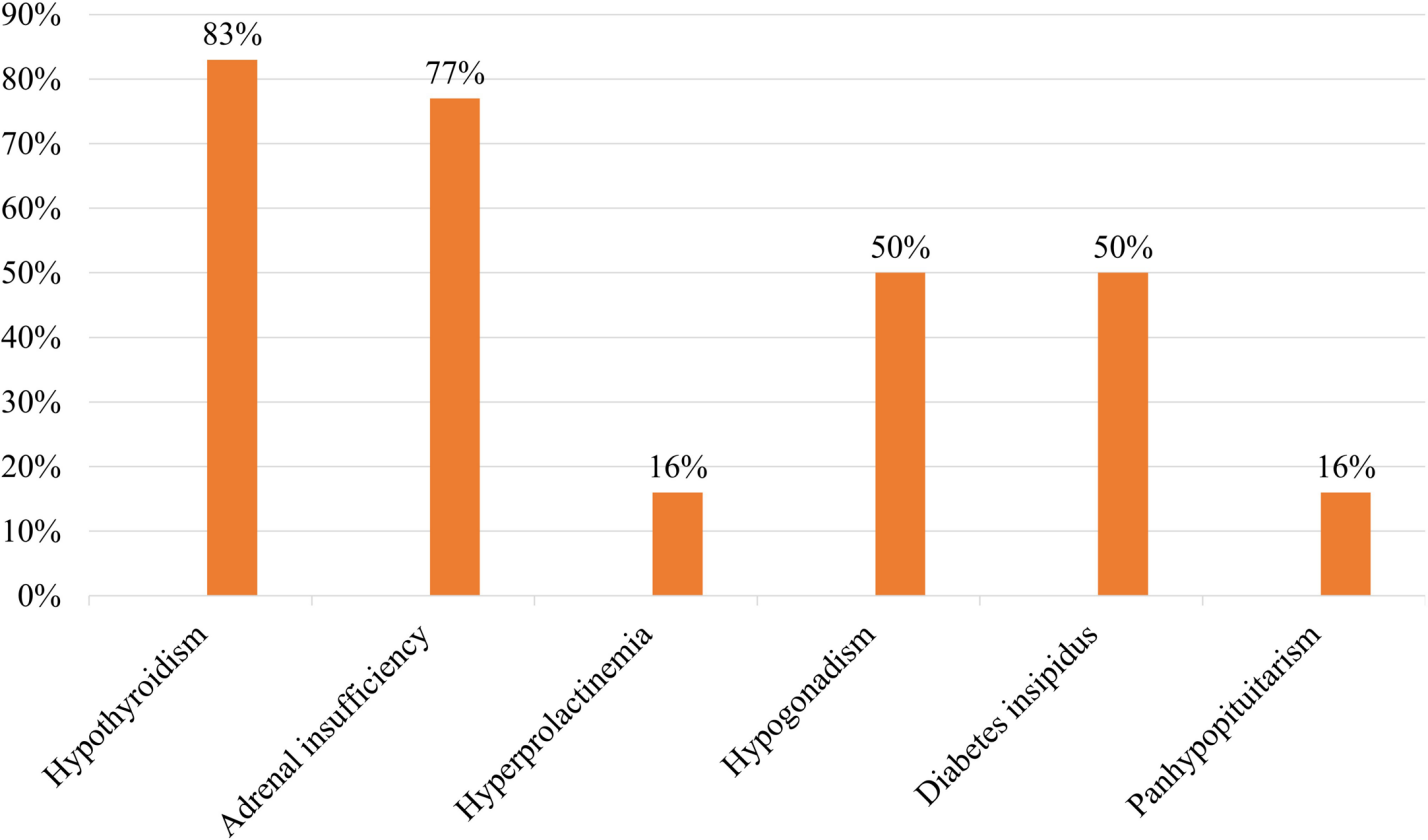
Hormonal abnormalities in patients with pituitary infiltration by DLBCL.

The symptoms of pituitary infiltration by DLBCL include both systemic B symptoms (such as weakness/fatigue, fever, night sweats, nausea/vomiting, and weight loss) and local symptoms from pituitary involvement. As shown in **Figure 4**, weakness/fatigue was the most common presenting Clinical feature. Hypothyroidism and adrenal insufficiency represented the most prevalent forms of endocrine hormone abnormalities (**Figure 5**). In our case, the patient presented with clinical manifestations including fatigue, weight loss, fever, and hormonal abnormalities such as hypothyroidism, adrenal insufficiency, growth hormone deficiency, and hypogonadism. The absence of hyperprolactinemia and diabetes insipidus was noted. This clinical picture is consistent with the typical presentation of the disease. It is worth noting that growth hormone deficiency often remains undetected.

Regarding the sequence of symptom onset between lymphoma and pituitary infiltration, some scholars have suggested that in the majority of patients, pituitary infiltration precedes the development of lymphoma (22). However, the two conditions may also manifest simultaneously, or pituitary involvement may occur subsequent to the diagnosis of lymphoma, which may be considered as pituitary metastasis. Given the concurrent presence of diffuse large B-cell lymphoma and pituitary involvement in this case, we classify this condition as pituitary infiltration rather than pituitary metastasis.

MRI lacks specific features for pituitary lymphoma infiltration. It may show lower signal intensity on both T1-weighted and T2-weighted images compared to pituitary adenomas, and demonstrates heterogeneous enhancement following contrast administration (23). Proton magnetic resonance spectroscopy (1H-MRS) is a novel imaging technique that aids in differentiating primary brain tumors from lymphoma, demyelinating lesions, and infectious lesions. Cerebrospinal fluid (CSF) cytology/flow cytometry can serve as a minimally invasive diagnostic approach (19). The diagnosis is primarily established based on cumulative evidence, such as a known history of DLBCL, suggestive MRI/PET/CT activity, hormonal abnormalities, and a dramatic response to DLBCL-specific chemotherapy. However, the gold standard for diagnosis remains histopathological biopsy. Given the invasive nature and technical challenges associated with pituitary biopsy, it is seldom performed in clinical practice. In addition, many patients have already received glucocorticoid treatment by the time pituitary infiltration is highly suspected. Preoperative corticosteroid administration may influence pathological findings in lymphoma patients and reduce the diagnostic yield of biopsies, likely due to the profound pro-apoptotic effects of corticosteroids on lymphocytes (24).

**Figure 6:**
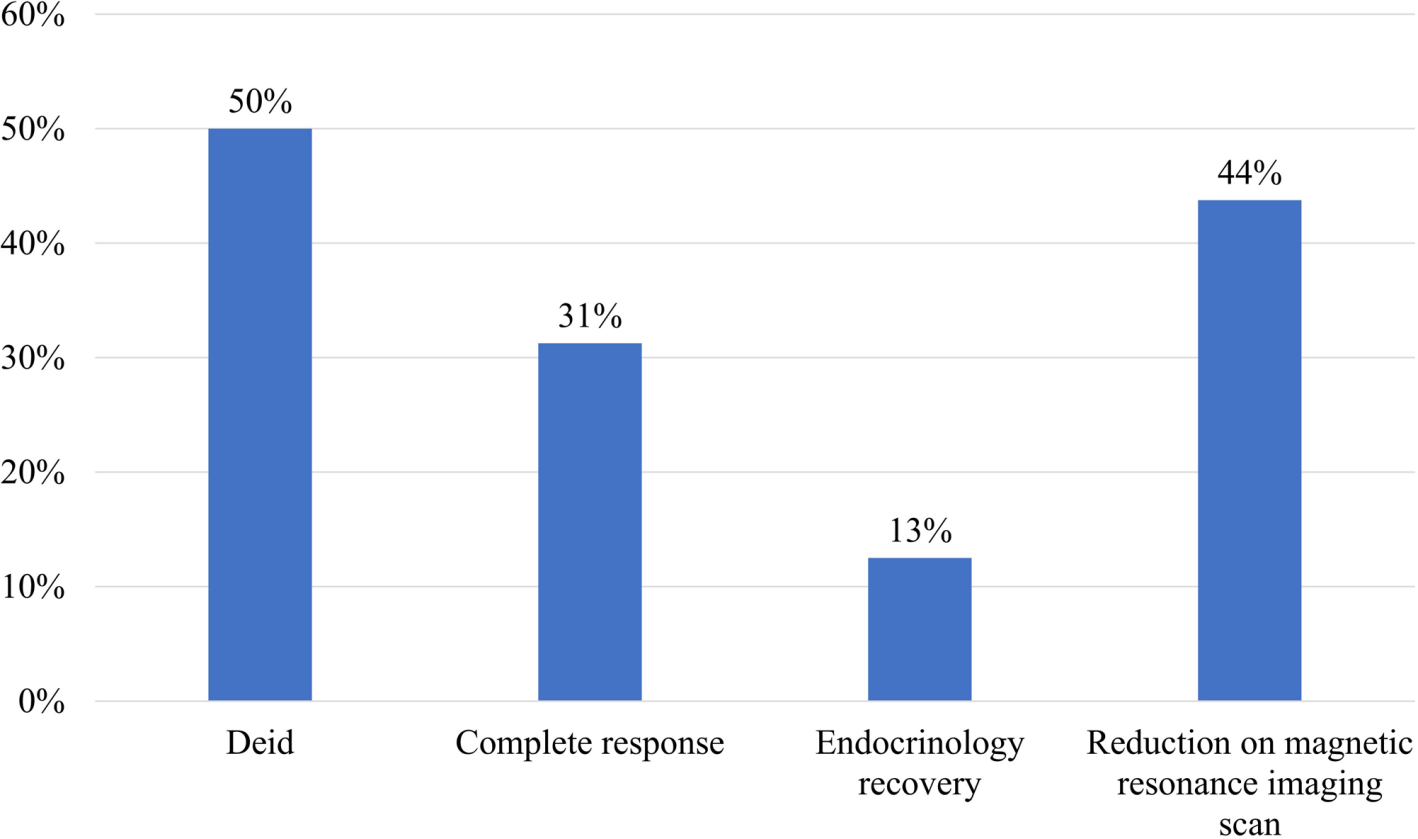
Prognosis of patients with pituitary infiltration by DLBCL.

Usually, the prognosis is poor once the pituitary gland is infiltrated by lymphoma (25). Mortality was reported in 44% of patients, often attributed to sepsis (26). The therapeutic focus involves hormone replacement therapy and systemic chemotherapy utilizing agents capable of crossing the blood-brain barrier (19). The chemotherapy regimen primarily consisted of rituximab plus CHOP (cyclophosphamide, doxorubicin, vincristine, and prednisone) (4). In our analysis, the mortality rate for pituitary infiltration by DLBCL was 50%, with survival times ranging from 5 days to 4 years (**Figure 6**). **Figures 4**–**6** were derived from a mini systematic review without a standalone methodology section. In this case, the patient’s outcome was favorable.

## Conclusion

Diagnosing pituitary infiltration by DLBCL is challenging due to nonspecific presentations. Re-evaluation should be considered for cases with poor treatment response or multisystem involvement to establish an accurate diagnosis.

## Limitation

The patient was already on glucocorticoid therapy at the time of baseline cortisol measurement.

## Data Availability

The original contributions presented in the study are included in the article/supplementary material. Further inquiries can be directed to the corresponding author.
